# Functional Impairment of Myeloid Dendritic Cells during Advanced Stage of HIV-1 Infection: Role of Factors Regulating Cytokine Signaling

**DOI:** 10.1371/journal.pone.0140852

**Published:** 2015-10-22

**Authors:** Meenakshi Sachdeva, Aman Sharma, Sunil K. Arora

**Affiliations:** 1 Department of Immunopathology, Postgraduate Institute of Medical Education & Research, Chandigarh, India; 2 Department of Internal Medicine, Postgraduate Institute of Medical Education & Research, Chandigarh, India; University Hospital Zurich, SWITZERLAND

## Abstract

**Introduction:**

Severely immunocompromised state during advanced stage of HIV-1 infection has been linked to functionally defective antigen presentation by dendritic cells (DCs). The molecular mechanisms behind DC impairment are still obscure. We investigated changes in DC function and association of key regulators of cytokine signaling during different stages of HIV-1 infection and following antiretroviral therapy (ART).

**Methods:**

Phenotypic and functional characteristics of circulating myeloid DCs (mDCs) in 56 ART-naive patients (23 in early and 33 in advanced stage of disease), 36 on ART and 24 healthy controls were evaluated. Sixteen patients were studied longitudinally prior-to and 6 months after the start of ART. For functional studies, monocyte-derived DCs (Mo-DCs) were evaluated for endocytosis, allo-stimulation and cytokine secretion. The expression of suppressor of cytokine signaling (SOCS)-1 and other regulators of cytokine signaling was evaluated by real-time RT-PCR.

**Results:**

The ability to respond to an antigenic stimulation was severely impaired in patients in advanced HIV-1 disease which showed partial recovery in the treated group. Mo-DCs from patients with advanced HIV-disease remained immature with low allo-stimulation and reduced cytokine secretion even after TLR-4 mediated stimulation *ex-vivo*. The cells had an increased expression of negative regulatory factors like SOCS-1, SOCS-3, SH2-containing phosphatase(SHP)-1 and a reduced expression of positive regulators like Janus kinase(JAK)2 and Nuclear factor kappa-light-chain-enhancer of activated B cells(NF-κB)1. A functional recovery after siRNA mediated silencing of SOCS-1 in these mo-DCs confirms the role of negative regulatory factors in functional impairment of these cells.

**Conclusions:**

Functionally defective DCs in advanced stage of HIV-1 infection seems to be due to imbalanced state of negative and positive regulatory gene expression. Whether this is a cause or effect of increased viral replication at this stage of disease, needs further investigation. The information may be useful in design of novel therapeutic targets for better management of disease.

## Introduction

An adequate host immune response against a pathogen relies on a concerted action of both innate and adaptive immune systems. Increasing attention is being embarked to the host innate immune responses that can react to molecular ‘patterns’ rather than specific peptide sequences and can exert a rapid anti-HIV activity without requiring an adaptive process [1].

Among cells of the innate immune system, myeloid dendritic cells (mDCs) are potent antigen presenters capable of inducing robust immunity and establishing immunological memory [2]. Down-modulation of DC maturation capabilities is commonly adopted by many pathogens including HIV-1 to evade immune responses and enhance their survival in the host [3].

In advanced stage of HIV-1 infection, patients become more vulnerable to opportunistic infections concurrent with progressive loss of CD4+ T-cells compared to those in early or intermediate stages [4]. Studies in macaques have shown that loss of DCs at virus set point predicts faster disease progression whereas an increase in their numbers predicts long term non-progression [5]. Thus, the functional defects of DCs linked with disease progression, could be considered at least in part responsible for susceptibility to AIDS-related illness in the advanced stage.

Despite this information, functional status of DCs during HIV-1 infection still remains inconclusive with majority of reports describing a defective state [6–8], some indicating partial reconstitution of DC functions in patients receiving antiretroviral therapy (ART) [9], while few others indicate a normal phenotype during HIV disease [10–12]. Hence a comprehensive analysis of DC maturation capabilities at different stages of HIV-1 infection becomes imperative so as to harness this important immune component in order to define novel strategies for better management of this debilitating disease.

Although the molecular mechanisms behind DC impairment are still not clear, yet growing evidence from *in-vitro* studies suggests role of both host-related genetic as well as the virus-mediated acquired factors [13–16]. The repertoire of cytokines in the microenvironment as well as secreted by the DCs has a crucial role in determining the fate of naïve T cells [17]. A dysregulation in cytokine signaling could be speculated in rendering the DCs defective during HIV-1 infection. Among the factors regulating cytokine signaling, a member of suppressor of cytokine signaling (SOCS) protein family, SOCS-1 is known to play a major regulatory function in macrophages and DCs because a large number of cytokines transduce their extracellular signals to the nucleus via the signal transducers and activator of transcription (STAT) proteins and the duration or intensity of cytokine induced signal is under feedback regulation of SOCS-1 protein [18,19]. Besides SOCS-1, other members of same family like SOCS-3 also negatively regulate the action of certain cytokines and STAT transcription factors [20]. Another regulator of cytokine signaling is SH2-containing phosphatase (SHP)-1 protein [21]. This phosphatase is constitutively expressed and can attenuate cytokine signal transduction by dephosphorylating signaling intermediates such as Janus kinases (JAK) and its receptor. Members of the protein inhibitors of activated STATs (PIAS) are also constitutively expressed in DCs and can attenuate signal transduction by repressing STAT activity [22]. Moreover DC maturation is promoted by the Nuclear factor kappa-light-chain-enhancer of activated B cells (NF-κB) which then mediates the downstream expression of various cytokines resulting in the induction of effector immune responses [23]. In this study, we have investigated the role of some key intrinsic factors regulating cytokine signaling to delineate the mechanisms causing functional impairment of DCs during HIV-1 infection. Our findings suggest that the HIV-1 infected patients, particularly in the advanced stage had an imbalanced expression of negative and positive regulators of cytokine signaling leading to profound negative effect on JAK-STAT or TLR-NF-κB pathways exerting inhibitory effects on DC function.

## Materials and Methods

### Ethical statement

The study was approved by the Institutional Ethics Committee of Post Graduate Institute of Medical Education & Research (PGIMER), Chandigarh, India and an informed written consent was obtained from all the subjects before drawing the blood samples.

### Study Groups

This cross-sectional study was performed on 92 HIV-1 infected patients (61 males, 31 females) visiting the Integrated Counseling and Testing Center (ICTC), Department of Immunopathology and ART clinic at the PGIMER, Chandigarh, India. The clinical characteristics of patients in different study groups are presented in **Table 1.** Of the recruited subjects, 56 were ART-naive and were subdivided into 2 groups based on their CD4+ T-cell counts: 23 patients in advanced stage of disease with CD4+ T-cell counts<250 cells/μL (mean±SD = 183±71), and 33 patients in early stage of disease with CD4+ T-cell counts>250 cells/μL (mean±SD = 551±174). In a separate group, 36 patients (mean±SD = 456±271 cells/μL) receiving triple combination ART consisting of Lamivudine, Zidovudine/Stavudine with Nevirapine/Efavirenz or Lamivudine, Tenofovir with Lopinarivir/Ritonavir for at least 1 year were also recruited. Patients with Tuberculosis and other chronic infections (Hepatitis C and B) were excluded from the study. The patient groups were compared with 24 healthy HIV-1 negative volunteers as controls. To understand the effect of ART on maturation dynamics of DCs, we also performed a longitudinal analysis of DC phenotype in 16 patients before and 6 months after ART initiation. Fifteen mL of peripheral venous blood was collected in heparinized vacutainer tubes (BD Biosciences, San Jose, CA, USA) for analysis from each subject.

**Table 1:** Clinical characteristics of individuals in each group.

**Study groups**	**Number**	**Males/Females**	**Mean Age (Years)**	**Mean CD4+ T-cell count (cells/μL)**	**Months of ART duration (range)**	**ART regime**
ART-naïve<250	23	19/4	31	183 ± 71	NA	NA
ART-naïve>250	33	17/16	34	551 ± 174	NA	NA
On ART	36	27/11	35	456 ± 271	38 (12–72)	A or B
HC	24	10/14	32	NA	NA	NA

A Lamivudine, Zidovudine/Stavudine with Nevirapine or Efavirenz

B Lamivudine, Tenofovir with Lopinarivir/ Ritonavir, NA not applicable

### Analysis of phenotypic markers on mDCs

The following antibody conjugates were used for phenotype analysis: Lineage cocktail-fluorescein isothiocyanate [FITC], anti-CD11c-phycoerythrin [PE]-Cy5, anti-HLA-DR-allophycocyanin [APC], anti-CD83, CD80, CD40, CD86-PE, anti-CD197 (CCR7)-BD Horizon™ V450 (BD Biosciences, San José CA, USA) and anti-CD198 (CCR8) PE (R&D Systems, Minneapolis, MN, USA). Appropriate isotype control antibodies were also used. The expression of all surface molecules on mDCs was assessed at 5 hours after *ex-vivo* stimulation with TLR4 agonist, Lipopolysaccharide (LPS) (Sigma-Aldrich, USA), as previously described [24]. For this assay, 90μl of whole blood was aliquoted per tube into pre-labelled 12X75mm Falcon^TM^ polystyrene test tubes (BD Biosciences, USA) and stimulated with LPS with effective concentration of 500ng/ml in RPMI-1640 medium consisting of 5% pooled human AB serum, 1% HEPES buffer, 0.2 mM L-glutamine, 100 units/ml of penicillin and 100 μg/ml of streptomycin. The unstimulated tube received 10μl of medium only (control).

The tubes were incubated at 37°C in a 5% CO_2_ humidified atmosphere for 5 hours at a 5° slant. Following incubation, surface staining was performed by adding fluorochrome-conjugated antibodies to the respective tubes. A fixed number of cells were acquired for each sample (300,000 total events) on flowcytometer, FACS ARIA (Becton Dickinson, USA) and data analyzed using FACSDiva software (BD Bioscience, San José, CA, USA).

### Generation of monocyte-derived cells (mo-DCs) for functional Assays

Monocytes were prepared from peripheral blood mononuclear cells (PBMCs) using the plate adherence method whereby PBMCs were placed in media containing RPMI-1640, 1% HEPES buffer, 0.2mM L-glutamine and antibiotics for 2 hours in T25 culture flasks (BD Falcon) at 37°C with 5% CO_2_. Monocytes have a characteristic property to adhere to the plastic surface, therefore, non-adherent cells were washed off with warm plain RPMI from flasks and the adherent cells were incubated further in differentiation medium.

Adherent monocytes were cultured in dendritic cell culture medium (DCCM) consisting of RPMI-1640 supplemented with: 2 mM L-glutamine, 5 mM HEPES buffer, 100 U/ml penicillin and 100 μg/ml streptomycin, 10% fetal bovine serum (Sigma-Aldrich, USA), along with dendritic cell growth factors 20 ng/ml recombinant human GM-CSF and 20 ng/ml recombinant human IL-4 (both from Peprotech, Israel), for 6 days in a humidified incubator (Thermo Forma, USA) at 37°C and 5% CO_2_. Half medium exchange was performed every 2 days with fresh DCCM. After 6 days of culture, the differentiated mo-DCs were checked for the purity by staining them with HLA-DR APC and CD11c PE-Cy5.5 (average percentage: 60–70%) followed by culturing with maturation cocktail [consisting of DCCM + LPS (500 ng/ml)] for 48 hours to induce DC maturation *ex vivo*. Simultaneously the control cells were also maintained on DCCM without any supplementation of LPS which are expected to remain as immature DCs, for comparative analysis. At the end of 48 hours, unbound and loosely adherent DCs were washed, collected and various functional assays were performed.

### Endocytosis Assay and Mixed Lymphocyte Reaction (MLR)

To assess the endocytosing capacity, LPS-stimulated mo-DCs were allowed to uptake 1mg/ml FITC-dextran (Sigma-Aldrich, USA) at 37°C, or at 4°C to determine background non-specific binding. The uptake of FITC-dextran was measured in terms of fold change in median fluorescence intensity (MFI) (ratio of MFI at 37°C to MFI at 4°C) as analyzed on flow cytometer (FACS Calibur, Becton Dickinson, USA).

To assess the allostimulatory capacity, LPS-treated mo-DCs were co-cultured with allogenic PBMCs from a healthy control (1:10) for 3 days. Lymphocyte proliferation was assessed by Dye Dilution Assay using carboxyfluoresceinsuccinimidyl ester (CFSE) on a flowcytometer (FACS Calibur). The stimulation index (SI) was calculated by dividing the percentage of dividing lymphocytes co-cultured with DCs by those cultured alone.

### Analysis of Inflammatory Cytokines

On day 6 of culturing, around 200,000 cells were cultured in 2ml DCCM in 6-well plates (BD falcon, USA) and stimulated with 500ng/ml of LPS for 2 days. At the end of day 8 of culture, the levels of cytokines: IL-1β, IL-6, IL-8, IL-12, IL-10 and TNFα were measured in culture supernatants from the LPS stimulated mo-DCs using Cytometric Bead Array (CBA Human Inflammation Kit, BD Biosciences, USA) as per manufacturer’s instructions.

### Gene expression Analysis

To further elucidate the mechanism behind DC impairment during HIV-1 infection, we analyzed the expression of suppressor of cytokine signaling (SOCS)-1 by quantitative real-time RT-PCR. At the end of day 8 of culture, RNA extracted from LPS stimulated mo-DCs using RNeasy mini kit (Qiagen, Hilden, Germany) was reversed transcribed to cDNA using Revertaid first strand cDNA synthesis kit (MBI Fermentas, USA). PCR was carried out with SYBR Green (Roche, CA, USA) incorporation using the primer, *forward* 5’-TTCGCCCTTAGCGTGAAGATGG-3’ and *reverse* 5’-TAGTGCTCCAGCAGCTCGAAGA-3’ in a RT-PCR machine (Light cycler, Roche Diagnostics, Indianapolis, USA) and analyzed by comparing the Ct values after normalizing the data with the house keeping gene, β-actin [25]. The expression of other genes, SOCS-3, SH2-containing phosphatase (SHP)-1, Janus kinase (JAK)2, protein inhibitors of activated STATs (PIAS)-1, nuclear factor kappa-light-chain-enhancer of activated B cells (NF-κB)1 and the house keeping genes, β-actin and GAPDH was also assessed in few subjects, by a custom designed PCR array (Qiagen, Hilden, Germany) and analyzed using RT^2^ profiler PCR Array Data analysis software (SAB biosciences, Qiagen).

### SOCS-1 Gene silencing

Gene silencing of SOCS-1 was performed by adding lipofectamine complexed with 5nmol of siSOCS-1 RNA (Qiagen, Hilden, Germany) to mo-DCs, which were then stimulated with LPS (500ng/ml) for 48 hours. SOCS-1 mRNA inhibition was determined by comparisons of mRNA expression between siRNA treated groups versus non-silencing siRNA after normalizing with β-actin gene as per manufacturers recommendation. Culture supernatants were collected and analyzed for cytokines using Cytometric Bead Array (CBA Human Inflammation Kit, BD Biosciences, USA) as per manufacturer’s instructions. Surface expression of co-stimulatory molecules was assessed by flowcytometry. MLR was performed using siRNA treated mo-DCs of patients and PBMCs of a healthy control by CFSE dye dilution method.

### Statistical Analysis

All data were analyzed using GraphPad Prism software (version 5.0, GraphPad Software Inc., La Jolla, CA, USA). Data were expressed as mean ± standard deviation (mean ± SD). The Wilcoxon matched-pairs signed rank test was used for comparison of pre- and post- therapy data. For multiple comparison between groups, Kruskal-Wallis test and for treatments across multiple tests, Friedman test was used, given a P value less than 0.5, Dunn’s multiple comparison adjustment was done. Spearman’s *r* test was used to determine correlations between two variables. P<0.05 was considered statistically significant.

## Results

### HIV-1 patients in advanced stage have diminished mDC frequency and responded poorly to LPS-stimulation with partial reconstitution following ART

**Fig 1:**
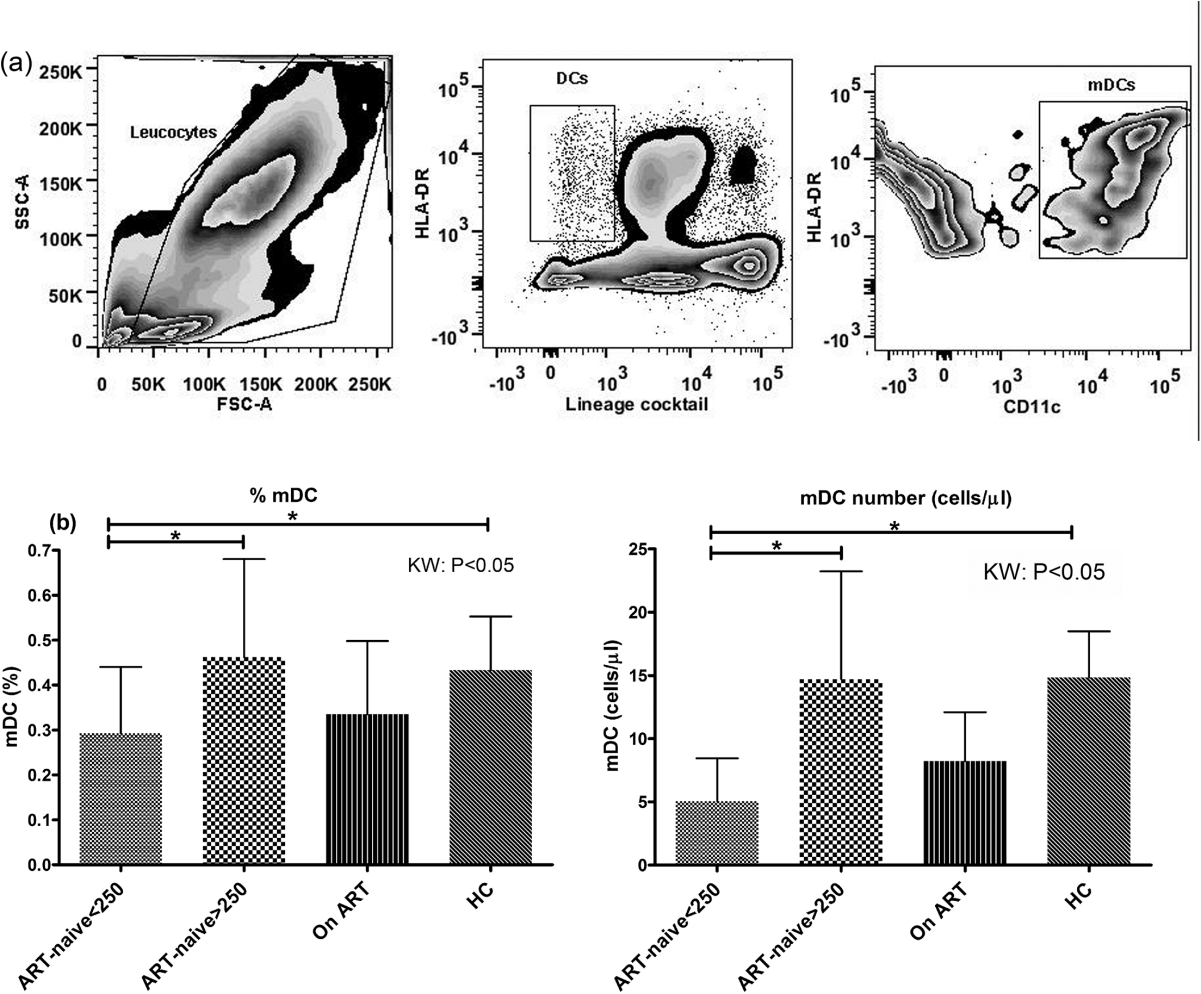
HIV-1 infected patients (CD4+ T-cell counts<250) have lower frequency of mDCs. (a) A representative picture showing strategy for gating mDCs in a healthy control subject wherein total leucocytes were gated for selection of DCs (Lineage-ve, HLA-DR+ve) for onward selection of myeloid DC cluster (CD11c+ve and HLA-DR+ve). (b) The percentage of mDCs were significantly lower in ART-naïve patients, CD4+ T-cell counts<250 cells/μL (n = 23) as compared to patients with CD4+ T-cell counts>250 cells/μL (n = 33) and HCs (n = 24) (p<0.05). (c) The absolute count of mDCs (cells/μL) was also sigificantly lower in ART-naïve patients, CD4+ T-cell counts<250 cells/μL as compared to patients with CD4+ T-cell counts>250 cells/μL and HCs (KW: p<0.05). Statistical analysis was performed using Kruskall-Wallis (KW) test for comparisons between multiple groups with pairwise comparisons using Dunn's multiple comparison adjustment for an overall p value<0.05. Data are represented as mean ± SD. *, P<0.05

Overall, the HIV-1 infected patients had low percentage of circulating mDCs, defined as lineage (CD3, CD14, CD16, CD19, CD20 and CD56) negative cells co-expressing CD11c and HLA-DR (Fig 1A). We observed an overall significant difference between the different study groups (p<0.05 by Kruskall-Wallis test follwed by Dunn's multiple comparison) like patients with CD4+ T-cell counts<250cells/μL having significantly lower frequency (mean±SD = 0.29±0.15) than healthy controls (HC) (0.43±0.12) as well as subjects with CD4+ T-cell counts>250cells/μL (0.46±0.22) (Fig 1B). A similar trend was observed for the mDC absolute count (cells/μL) in which the ART-naive patients (CD4+ T-cell counts<250) had significantly lower count of circulating mDCs (mean±SD = 5±3) than HCs (15±4) as well as subjects with CD4+ T-cell counts>250cells/μL (15±8) (p<0.05) (Fig 1C). The treated group however did not show significant improvement over the untreated ones.

**Fig 2:**
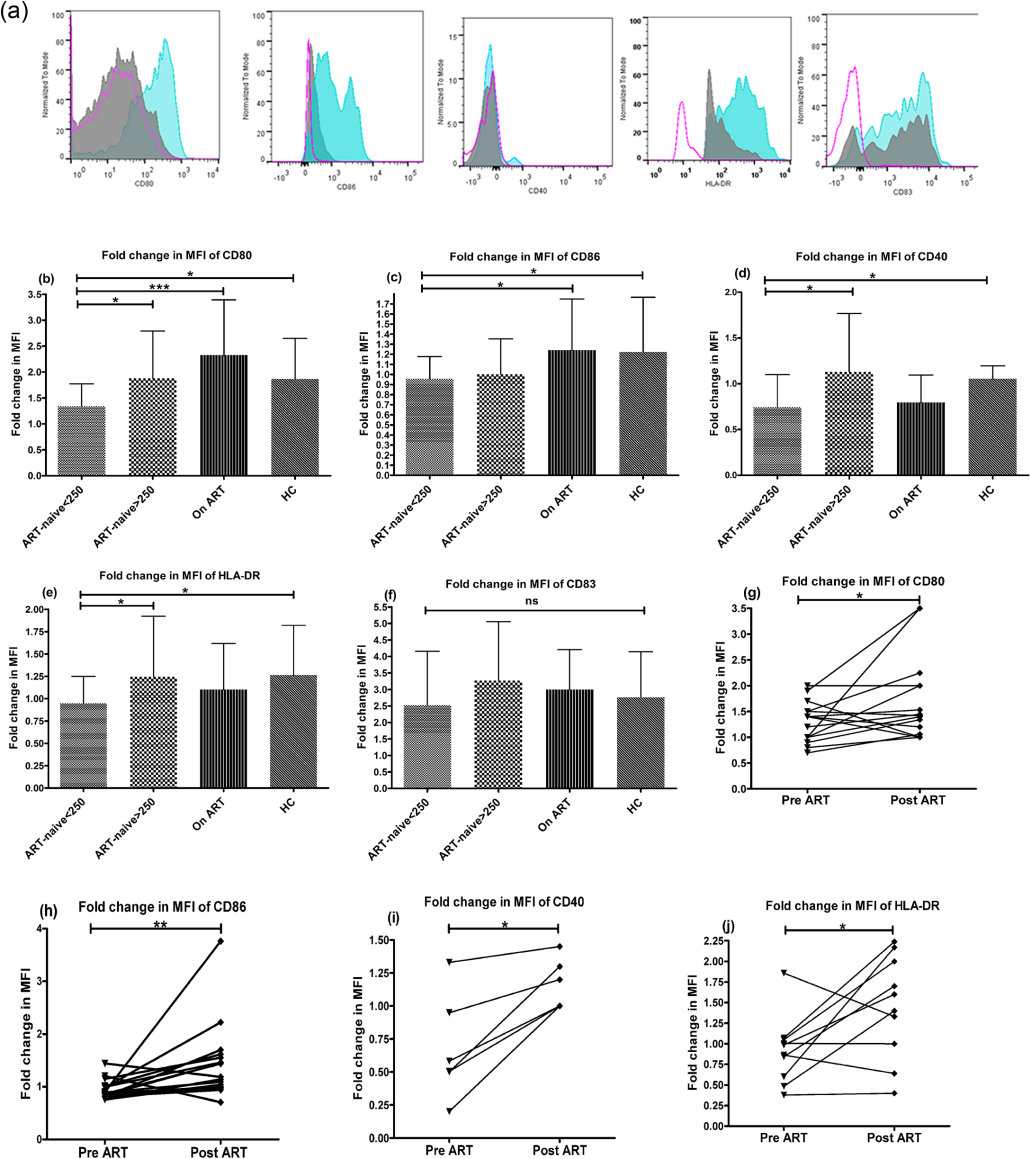
Reduced responsiveness of mDCs of ART-naive patients (CD4+ T-cell counts<250 cells/μL) to LPS-stimulation. The ability of circulating mDCs to respond to TLR stimulation was assessed using a whole blood DC assay by their abilities to upregulate costimulatory molecules CD80, CD86, CD40, maturation marker CD83 and HLA-DR in response to 5-hour stimulation with LPS (500ng/ml) by flowcytometry in 23 ART-naive patients in advanced stage of disease; 33 patients in early stage of disease, 36 patients on ART and 24 HCs. (a) A representative picture for all markers in a HC subject is shown. The open pink histogram shows the isotype control, solid grey histogram, the unstimulated control and solid blue histogram, the stimulated cells. The fold change in MFI, as a measure of change in expression level of each marker of stimulated mDCs was calculated over the unstimulated mDCs. The HIV-1 infected patients in advanced stage (CD4+ T-cell counts<250) had significantly lower upregulation of (b) CD80, (c) CD86, (d) CD40 and (e) HLA-DR as compared to HCs (p<0.05). (f) The expression of CD83 was not statistically different between the HIV-infected groups and the HCs. Statistical analysis was performed using Kruskall-Wallis (KW) test for comparisons between multiple groups with pairwise comparisons using Dunn's multiple comparison adjustment for an overall p values <0.05. Longitudinal analysis of 16 patients pre- and post- 6 months of ART was done by Wilcoxon matched-pairs signed rank test and revealed a significant increase in the expression of CD80, CD86, CD40 (n = 6) and HLA-DR (g to j) but not for CD83. Data are represented as mean ± SD. *, P<0.05; **, P<0.01; ***, P<0.001; ns, not significant.

The maturation potential of circulating mDCs in response to external stimuli was assessed by *ex-vivo* stimulation with TLR4 agonist, Lipopolysaccharide (LPS) (representative overlay flowhistograms in Fig 2A). The mDCs of patients in advanced stage of HIV disease were not able to upregulate the co-stimulatory markers, as indicated by a significantly lower fold change in (mean±SD) MFI of CD80(1.3±0.43), CD86(0.9±0.2), CD40(0.7±0.3) and HLA-DR(0.97±0.29) as compared to HCs(2±0.7, 1.22±0.5, 1±0.1, 1.27±0.5 respectively) (p<0.05) (Fig 2B to 2E), while no significant differences among various study groups was observed for maturation marker, CD83. The effect of therapy on functional improvement of mDCs was notable in patients receiving ART with higher expression of CD80 and CD86 versus ART-naive individuals with low CD4 numbers (p<0.05) (Fig 2B and 2C). Importantly, longitudinal assessment of patients prior to and after 6 months of ART, mDCs demonstrated increased responsiveness to TLR stimulation post-therapy with upregulation of co-stimulatory molecules, (mean±SD, 1.3±0.14 vs. 2±0.2 for CD80; 0.9±0.06 vs. 1.6±0.2 for CD86; 0.7±0.16 vs. 1.1±0.07 for CD40 and 0.92±0.11 vs. 1.5±0.18 for HLA-DR) (p<0.05) (Fig 2G to 2J) except for CD83. These results indicate that patients in advanced stage of disease with CD4+ T-cell counts below 250 cells/μL had deficit frequency of circulating mDCs and defective DC maturation potential in response to external stimuli with only partial reconstitution of DC functions one year post-ART.

### Defective expression of homing receptors on mDCs during advanced HIV-1 disease

**Fig 3:**
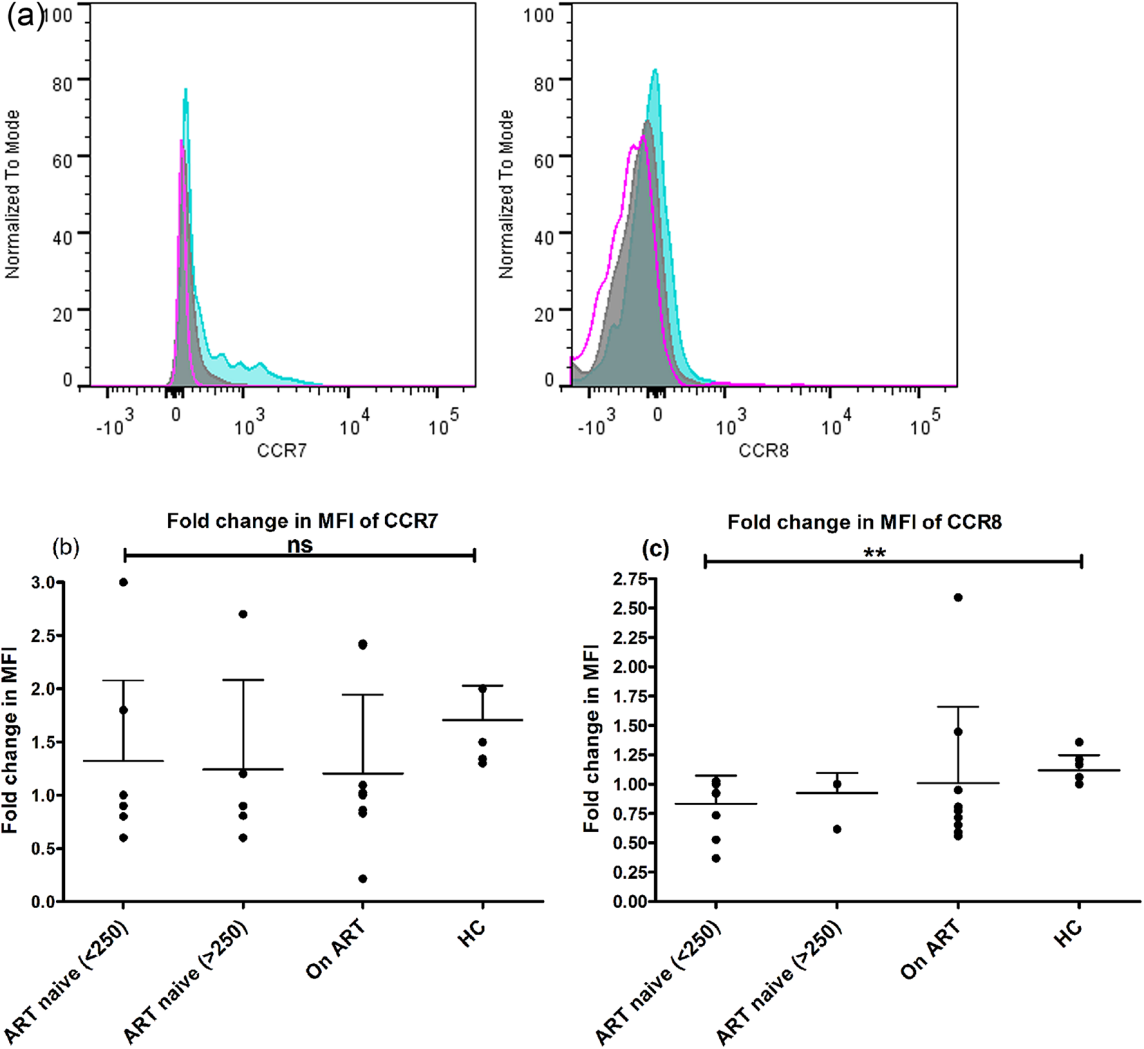
Defective phenotype of mDCs for migration from periphery to lymphoid tissues. (a) A representative flowcytogram of a healthy control subject showing CCR7 and CCR8 expression on mDCs. The open pink histograms shows the isotype control, solid grey histogram shows the unstimulated control and solid blue histogram shows the stimulated control. The ability of mDCs to upregulate chemokine receptors CCR7 (b) and CCR8 (c) in response to a 5-hour stimulation to LPS (500ng/ml) by flowcytometry in ART-naive patients with CD4+ T-cell counts<250 cells/μL (n = 9) and CD4+ T-cell counts>250 cells/μL (n = 5) and compared with patients on ART with CD4+ T-cell counts>350 cells/μL (n = 9). The patients in late stage of HIV had significantly lower ability to up-regulate CCR8 (p<0.05) as compared to HCs. Data are represented as mean±SD. **, P<0.01; ns not significant.

We have analyzed the expression of chemokine receptors CCR7 and CCR8 (representative picture in Fig 3A) in response to LPS in ART-naive patients with CD4+ T-cell counts<250 cells/μL (n = 9) and CD4+ T-cell counts>250 cells/μL (n = 5) and compared with patients on ART with CD4+ T-cell counts>350 cells/μL (n = 9). The mDCs from patients in advanced stage of disease showed diminished capacity to up-regulate CCR7(mean±SD: 1.3±0.7 vs. 2±0.34) and CCR8(0.8±0.2 vs. 1.1±0.1) although statistically non-significant as compared to HCs with statistical significance only for CCR8 (p<0.05) (Fig 3B and 3C). This observation suggests that migration of DCs may be partially impaired in these patients.

### Reduced antigen presentation and lymphocyte proliferation by mo-DCs of ART-naive patients in advanced stage of disease

**Fig 4:**
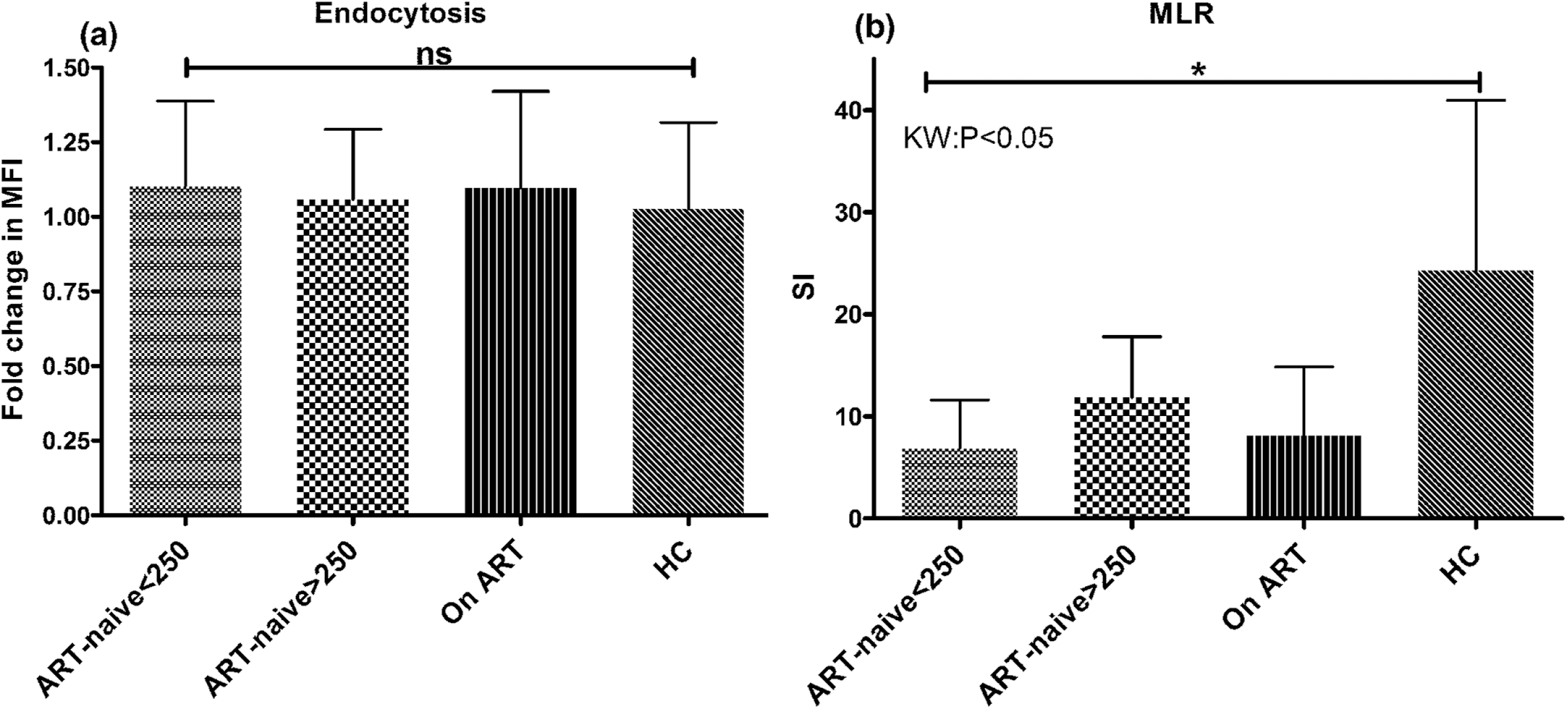
(a) Retained endocytosis ability of mo-DCs post LPS-stimulation. The fold change in the uptake of FITC-dextran as a measure of endocytosis was calculated in mo-DCs post-LPS stimulation (500ng/ml) for 2 days by flowcytometry in 23 ART-naive patients in advanced stage of disease; 33 patients in early stage, 36 patients on ART and 24 healthy controls. The mo-DCs from patients in advanced stage depict immature phenotype of mo-DC post-LPS stimulation as indicated by retained endocytosis of antigen even after maturation stimulus as compared to HCs although no significant differences were observed among various study groups. **(b) Allo-stimulation of healthy lymphocytes by mo-DCs of various patient groups.** The ability of mo-DCs to induce lymphocyte proliferation was evaluated by CFSE dye dilution method in ART-naive patients in advanced stage of disease (n = 9) and early stage of disease (n = 5) and the stimulation index was compared with patients on ART with CD4+ T-cell counts>350 cells/μL (n = 9). Mo-DCs of patients in advanced stage of HIV disease had significantly lower SI as compared to HCs (p<0.05). Data are represented as mean ± SD. *, P<0.05.

In order to further confirm the maturation status post-TLR stimulation, we assessed the endocytosing capability of mo-DCs using FITC conjugated dextran uptake assay. We observed no significant difference among the HIV-infected groups, however the mo-DCs from patients in advanced stage of HIV disease retained higher endocytosing capacity post-stimulation as compared to HCs (mean±SD:1.2±0.2 vs. 1±0.29), although statistically insignificant (Fig 4A). Furthermore, the allo-stimulatory capacity of these mo-DCs, as measured by carboxyfluoresceinsuccinimidyl ester (CFSE) dye dilution method in a mixed lymphocyte reaction (MLR), was found to be significantly lower (mean±SD:7.5±4.0) as compared to HCs (28±16) (p<0.05) indicating a diminished ability to activate lymphocytes (Fig 4B). There was however no significant improvement in these parameters post 1-year of ART, suggesting slow reconstitution of DC functions as compared to CD4+ T-cell counts in these patients. These findings indicate a compromised antigen presenting capacity of DCs, particularly, during advanced stages of HIV disease.

### Reduced expression of inflammatory cytokines from mo-DCs of patients in advanced HIV-disease

**Fig 5:**
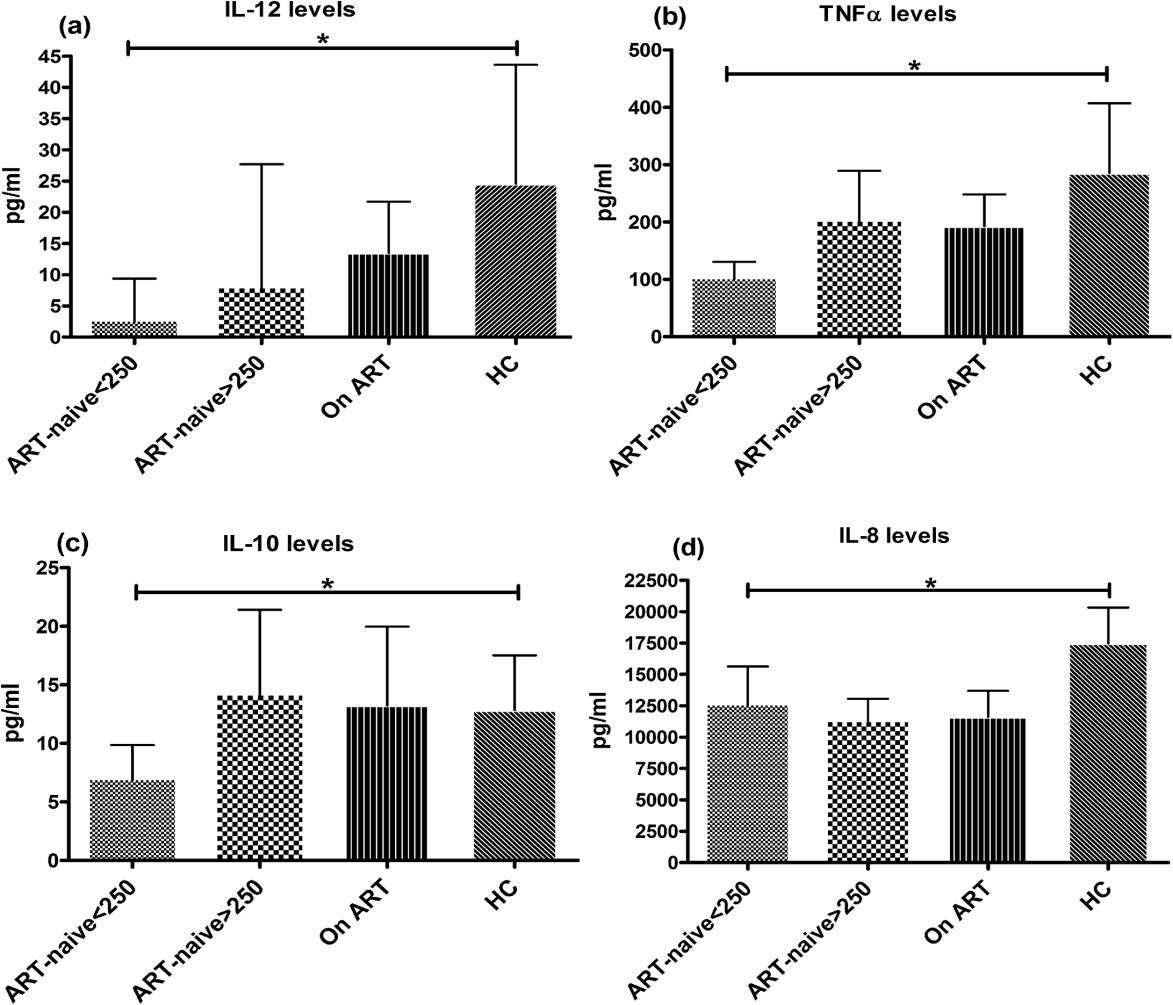
Cytokine secretion from mo-DCs post LPS-stimulation. Level of inflammatory cytokines (IL-12, TNFα, IL-10 and IL-8) was measured in the culture supernatants of mo-DCs after LPS-stimulation (500ng/ml) for 2 days by multiplex cytometric bead array (CBA) technique in 23 ART-naive patients in advanced stage of HIV disease; 33 patients in early stage, 36 patients on ART and 24 HCs and the results were expressed in pg/ml. The HIV infected patients in advanced stage of disease (CD4+ T-cell counts<250 cells/μL) had significantly lower secretion of (a) IL-12, (b) TNFα, (c) IL-10 and (d) IL-8 as compared to HCs (p<0.05). Patients with higher CD4+ T-cell counts had higher levels of IL-12, TNFα and IL-10, although statistically non-significant. Patients on ART did not depict significant increase in the levels of these cytokines. Data are represented as mean ± SD. *, P<0.05.

The levels of cytokines (IL-12, TNFα, IL-10, IL-8, IL-6 and IL-1β) were measured in the culture supernatants of mo-DCs post LPS-stimulation. Overall the secretion of cytokines IL-12(mean±SD: 2.5±7 vs. 24±85pg/ml), TNF*α*(100±148 vs. 285±544pg/ml), IL-10(7±13 vs. 13±20pg/ml) and IL-8(12518±13921 vs. 17470±12504pg/ml) were significantly lower in patients in advanced stage of disease as compared to HCs (p<0.05) (Fig 5). The levels of IL-12, TNF*α* and IL-10 were better in patients with CD4+ T-cell counts>250 cells/μL and also in treated group. Levels of IL-6 and IL-1β were lower but not significantly different from HCs (data not shown). These results further affirm previous findings showing down modulation of DC functions affecting cytokine production in advanced stage of disease.

### HIV-1 infected patients in advanced stage expressed higher levels of SOCS-1

**Fig 6:**
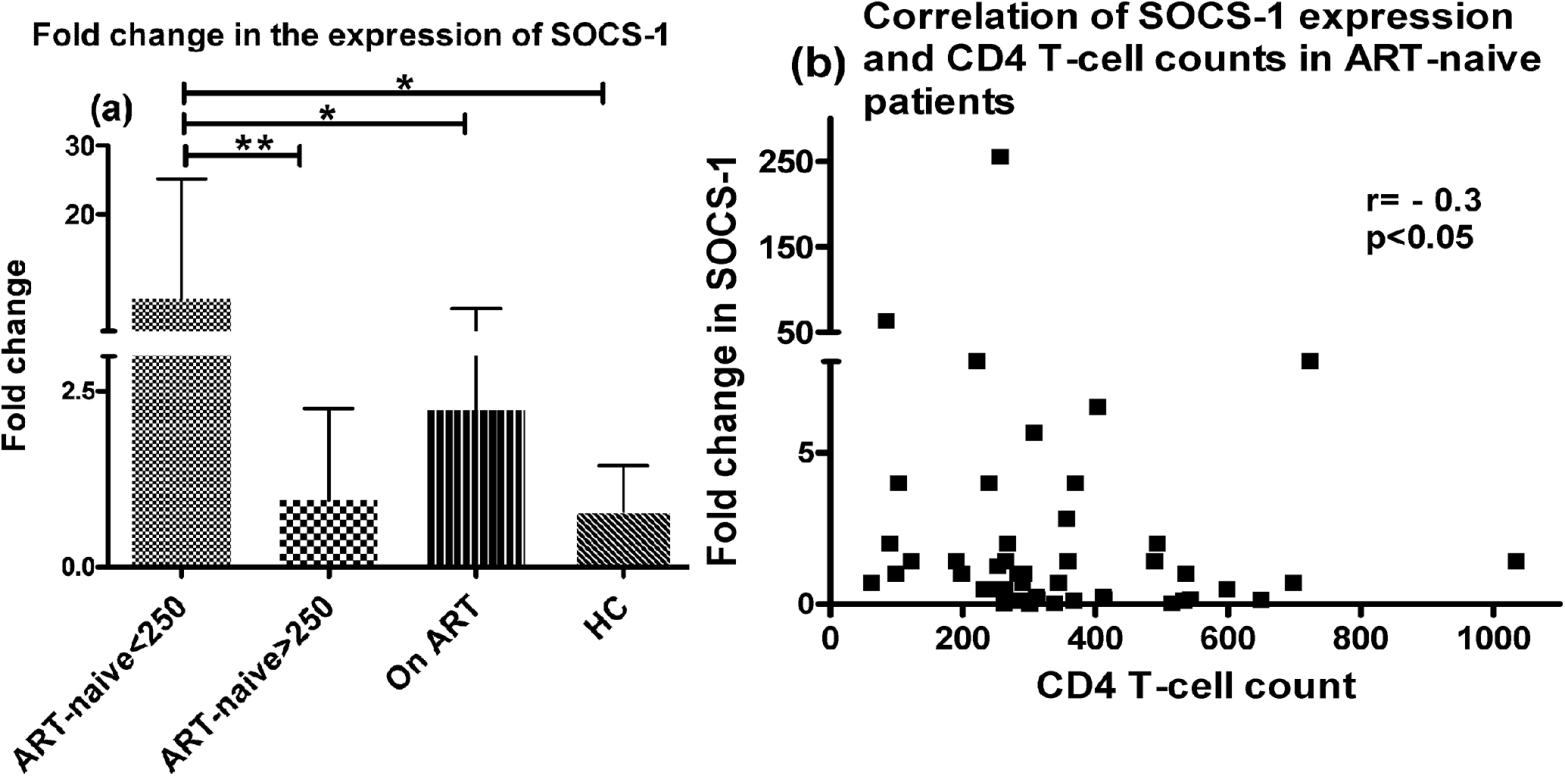
Increased expression of SOCS-1 in mo-DCs of ART-naive patients in advanced stage of HIV disease. The expression levels of SOCS-1 mRNA was measured relative to the house keeping gene β-actin post LPS-stimulation (500ng/ml) for 48hrs in 23 ART-naive patients in advanced stage of HIV disease; 33 patients in early stage, 36 patients on ART and 24 HCs by Real time PCR using SYBR Green chemistry. (a) Fold change in the levels were calculated over the unstimulated controls. Statistical analysis was performed using Kruskall-Wallis (KW) test for comparisons between multiple groups with pairwise comparisons using Dunn's multiple comparison adjustment for an overall p values <0.05. Patients with lower CD4+ T-cell counts (<250 cells/μL) had significantly higher expression than HCs and the patients with higher CD4+ T-cell counts (>250 cells/μL) (p<0.05). The patients on ART also had significantly lower levels of SOCS-1 expression as compared to ART-naïve patients in advanced stage of disease (p<0.05). (b) The SOCS-1 levels had significant inverse relationship with CD4+ T-cell counts among the ART-naive subjects (p<0.05). Data are represented as mean ± SD. *, P<0.05; **, P<0.01.

Since maturation of DCs is associated with production of various cytokines which are further regulated by regulatory factors like suppressor of cytokine signaling (SOCS) proteins, we interrogated whether upregulated expression of such factors during HIV-1 infection might have been the cause of low cytokine production and impaired functions. We observed a significantly higher expression of SOCS-1 in mo-DCs of patients in advanced stage of disease (<250 cells/μL) as compared to HCs (mean±SD: 8±17 vs. 0.8±0.6) (p<0.05) and patients in early stage of disease (0.95±1.3) (p<0.05) (Fig 6A). Interestingly the expression of SOCS-1 was found to be decreased (2.3±4) significantly (p<0.05) in the patients on ART after 1 year of treatment. We also observed a significant negative correlation between SOCS-1 expression and CD4+ T-cell counts among the ART-naive patients (Spearman correlation coefficient, r = -0.3, p<0.05) (Fig 6B).

### Factors facilitating DC impairment in HIV-1 infected patients in advanced stage

**Fig 7:**
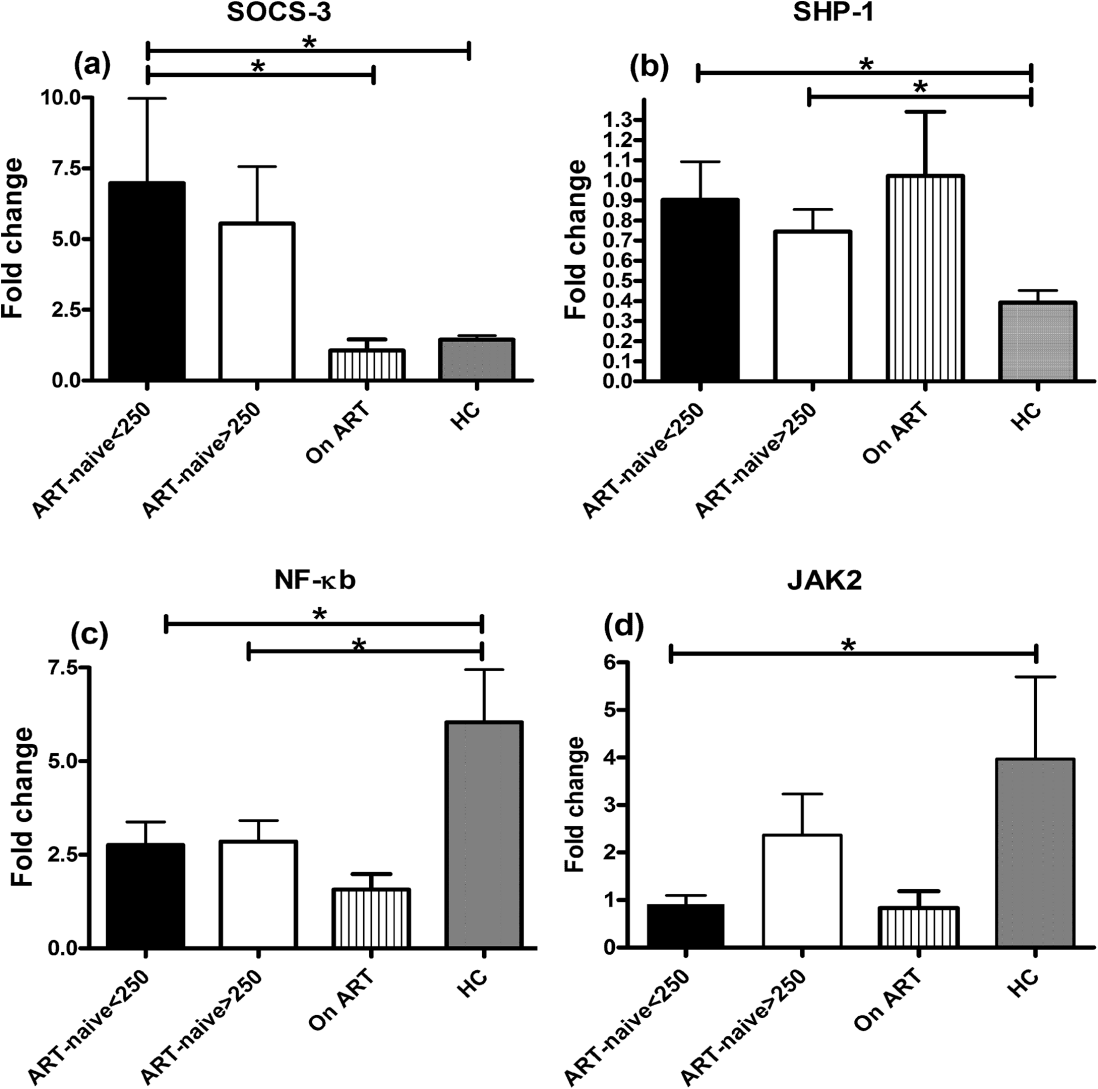
Expression levels of SOCS-3, SHP-1, NF-κB1 and JAK2 in different study groups. The fold change in the expression of SOCS-3, SHP-1, NF-κB and JAK2 was measured in ART-naive patients (n = 9) with CD4+ T-cell counts<250 cells/μL and patients with CD4+ T-cell counts>250 cells/μL (n = 9) along with HCs (n = 9) and patients on ART (n = 9). Kruskall-Wallis (KW) test was performed for comparisons between multiple groups with pairwise comparisons using Dunn's multiple comparison adjustment for an overall p values <0.05. (a) The expression of SOCS-3 was significantly higher in patients with CD4+ T-cell counts<250 cells/μL as compared to HCs and patients on ART (p<0.05). (b) The expression of SHP-1 mRNA levels were also significantly higher in patients in advanced HIV-disease as compared to HCs (p<0.05). (c) The expression of NF-κB1 and (d) JAK2 on the other hand was significantly lower (p<0.05) in this patient group compared to HCs. Data are represented as mean ± SD. *, P<0.05.

In order to delineate any other factors responsible for the functional impairment of DCs besides SOCS-1, in advanced stage of HIV-1 disease, we studied the expression levels of additional genes involved in regulation of cytokine synthesis, including, SOCS-3, SHP-1, PIAS-1, NF-κB1 and JAK2 in ART-naive patients, patients on ART and HCs. The data indicated higher expression of SOCS-3 in mo-DCs of patients with CD4+ T-cell counts<250 cells/μL as compared to HCs (mean±SD: 7±8 vs. 1.4±0.4) and patients on ART (1±1.1) (p<0.05) (Fig 7A), but no significant difference with patients having higher CD4 numbers (>250 cells/μL). Similarly, expression of SHP-1 gene was also significantly higher in patients with advanced HIV-disease as compared to HCs (mean±SD: 0.9±0.5 vs. 0.39±0.18) (p<0.05) (Fig 7B), but no significant difference in the expression levels of PIAS gene among the study groups was observed (data not shown). Importantly, NF-κB1, the major activator of cytokine pathway showed significantly lower expression in patients in advanced stage of disease (mean±SD: 2.7±1.8) as compared to HCs (6±4) (p<0.05) (Fig 7C). The expression level of JAK2 gene was also significantly lower in these patients as compared to HCs (mean±SD: 0.9±0.5 vs. 4±5) (p<0.05) (Fig 7D). These results thus, highlight the association of these factors in facilitating functional impairment of DCs in advanced stage of HIV-1 infection.

### Improvement in functions by SOCS-1 silencing in mo-DCs

**Fig 8:**
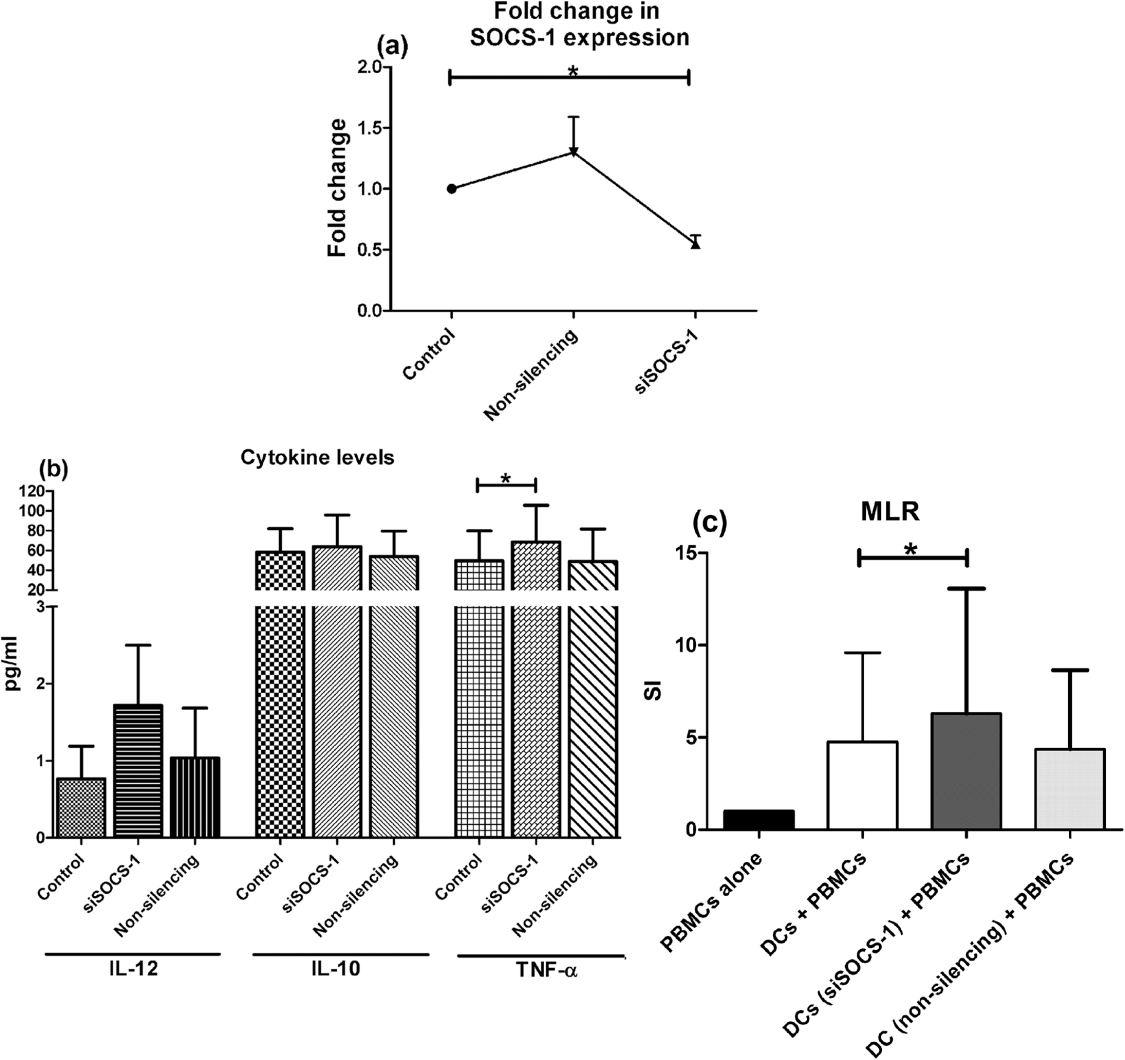
SOCS-1 silencing enhances cytokine production and stimulatory capacity of mo-DCs. Mo-DCs from 6 ART-naive patients in the advanced stage of HIV-1 infection were transfected with 5nmol siRNA to SOCS-1 gene for 48 hours. Cells treated with a scrambled non-silencing RNA or untreated cells served as control. Friedmann test was performed for comparisons between treated groups with pairwise comparisons using Dunn's multiple comparison adjustment for an overall p values <0.05. (a) Mo-DCs treated with siSOCS-1 had around 50% decrease in the expression of SOCS-1 gene which was significantly less than the control cells (p<0.05). (b) Culture supernatants were evaluated for the levels of inflammatory cytokines by CBA. The levels of IL-12, IL-10 and TNF-α were higher in SOCS-1 silenced mo-DCs which could reach statistically significant levels for TNF-α (p<0.05). (c).The allostimulatory capacity of SOCS-1 silenced mo-DCs was tested in co-cultures with PBMCs of a healthy individual by CFSE dye dilution assay. The SOCS-1 silenced mo-DCs induced a significantly higher allostimulation as compared to cells that had normal expression of SOCS-1. Data are represented as mean ± SD. *, P<0.05.

Further to validate our findings, we postulated whether SOCS-1 silencing could improve the functional state of defective mo-DCs in terms of enhanced cytokine production. For this, mo-DCs from 6 ART naive patients in advanced stage of HIV-1 infection (mean CD4+ T-cell counts: 225 ± 37) were treated with either siRNA to silence the expression of SOCS-1 gene or scrambled non-silencing RNA following LPS-stimulation for 48 hours. Cells not treated with siRNA served as control. The SOCS-1 expression was knocked down to 50% with 5nmol siRNA as compared to cells not treated with siRNA (Fig 8A). Culture supernatants from these cells were analysed for cytokine levels and we observed higher levels of IL-12 (mean ± SD: 1.7 ± 1.9 vs. 0.76 ±1.0 pg/ml), TNF-α (64 ± 90 vs. 50 ± 74 pg/ml) (p<0.05 by Friedman test) and IL-10 (64 ± 71 vs. 58 ± 53 pg/ml) in SOCS-1 silenced mo-DCs as compared to controls (Fig 8B). There was no change in the levels of these cytokines between controls and cells treated with non-silencing RNA.

Next, to assess whether there is any improvement in the allo-stimulatory capacity of mo-DCs post SOCS-1 silencing, we cocultured these treated and untreated mo-DCs with PBMCs from a healthy donor and observed a significantly stronger MLR response by SOCS-1 deficient mo-DCs as compared to controls (6.3 ± 6.7 vs. 4.7 ± 4.8) (p<0.05) (Fig 8C), although no significant change in the expression of co-stimulatory molecules was observed between SOCS1-silenced DCs versus controls (data not shown).

These findings suggest that an elevated SOCS-1 expression seems to be one of the causes of functional defects in terms of reduced cytokine secretion and diminished allo-stimulation by DCs in the advanced stage of HIV infection, which could be normalized to a great extent by inhibiting the expression of SOCS-1.

## Discussion

In advanced stage of HIV-1 infection, DCs are believed to be minimally infected [26], but continuous exposure to TLR ligands not only hampers their activation but also influences their maturation in response to other activation stimuli or infections. In this study, we have provided sufficient evidence that functional state of myeloid dendritic cells is more severely affected during advanced stage of HIV-1 infection when the CD4+ T-cell counts fall below 250 cells/μL, making these patients more prone to opportunistic infections. This functional impairment was reflected by reduced responsiveness to TLR ligands in terms of lower expression of co-stimulatory molecules, sub-optimal antigen presentation and diminution of cytokine production, all contributing to the already compromised state of immune system in these patients. Further, the data delineates the mechanisms of DC dysfunction indicated by increased expression of negative regulatory factors like SOCS-1, SOCS-3, SHP-1 and a lower expression of positive regulators such as JAK2 and NF-κB1.

Overall, a reduced frequency of circulating mDCs was observed in all patient groups including the treated group. This could be attributed to multiple factors including the presence of HIV that is directly responsible for killing the DCs, their redistribution to regional lymph nodes or due to lower rate of differentiation from progenitors [27]^,^[7,28–31].

The ability of DCs to mature in response to antigenic exposure is critical in optimal antigen presentation to naive T-cells for their activation and generation of an effective immune response [2]. There are contradictory reports regarding the functional status of DCs [7,10–12,29,30] owing to variations in the DC purification/culture procedures, ethnicity of study populations and the TLRs used for DC stimulation. Therefore, it was more prudent to gather information regarding functional state of DC in the HIV infected individuals at different stage of disease and subsequently look for the underlying mechanisms the dysfunctional state ensues. To closely mimic the *in vivo* status of DCs, the phenotypic evaluations were conducted on circulating mDCs in the peripheral blood of HIV infected individuals, while the functional studies were performed on the mo-DCs. Although the mo-DCs and mDCs represent two separate populations of DCs, with some distinct and some overlapping functions, yet our data indicate that the functioning of both the types of DCs are adversally affected only during advanced stage of HIV-1 infection.

Our findings support the defective TLR responsiveness of mDCs in ART-naive patients during advanced stage of HIV-infection showing lower upregulation of co-stimulatory and MHC molecules on stimulation ex vivo, suggesting poor maturation capability of these cells. These defects were only partial in patients in early stage of disease with near normal CD4+ T-cell counts showing normal up-regulation of CD80, CD40 and HLA-DR on LPS-stimulation, but a noticeable defect for other markers, like CD86 and CD83. Therefore it appears that DCs become defective gradually as the disease progresses eventually becoming defunct as the end stage culminates in clinically evident immune-deficiency. ART resulted in partial reconstitution of some of the functions, implying that once affected the DCs do not attain normal behavior as quickly as the recovery of CD4+ T-cell counts to normal levels. A better picture was obtained by longitudinal analysis of subjects on therapy, compared to cross-sectional evaluation. Here, the DCs exhibited a better response in terms of up-regulation of all DC markers post-ART except for CD83. However, shorter observation time of 6 months after the start of ART might be insufficient for reconstitution of DC functions.

Migration of DCs to regional lymph nodes after interaction with an antigen is a normal process during maturation of DCs and is mediated by expression of chemokine receptors [32]. In one study conducted in mice, DCs from a CCR7−/− animals failed to to migrate to draining lymph nodes, indicating the role of CCR7 in this process [33]. CCR8 is another chemokine receptor whose ligand is CCL1; its role is also defined in bone marrow derived mo-DCs during mobilization from skin to LNs along with CCR7 [34]. No defects in the expression of chemokine receptors were observed in a study on HIV infected patients in acute stage [27], whereas, we observed a deficiency in upregulation of chemokine receptors, CCR7 and CCR8 in patients in chronic advanced stage, suggesting that migration of mDCs may be impaired during this stage. Further, the defective maturation was also indicated in our study from retained endocytosing capacity even after maturation stimulus in contrast to a previous report, where immature DCs exhibited a higher endocytosis, that was almost completely lost following LPS-stimulation [16]. The maturation defective DC in the advanced stage of HIV disease also depicted suboptimal antigen presentation as indicated by poor allostimulation of lymphocytes, suggesting that poor-handling of bacterial infections in the late stage may also be attributed to functionally defective DCs in combination with low T-cell numbers. It was intriguing to note that the functional defects of DCs were reconstituted only marginally in patients on ART indicating a slow rate of reconstitution of functions post-treatment as compared to CD4+ T-cell numbers.

TLR signaling promotes DC maturation by activating mitogen-activated protein kinase and NF-κB, mediating expression of various cytokines, resulting in effective immune response [35,36]. We found suppressed IL-12 levels in all stages of HIV-1 infection, more prominent in patients with advanced stage of disease. Some authors have correlated this deficit with decreased CD40L expression on T-cells during advanced disease [37]. In another study, exposure of mo-DCs to plasma from untreated patients suppressed IL-12 and impaired Th1 responses [38]. We also found lower levels of TNFα, IL-10 and IL-8 in advanced stage and our results corroborated the findings of another study reporting reduced IL-8, IL-6, and TNFα in HIV-1 patients [6].

The published literature acknowledges the role of SOCS family of proteins in cytokine regulation. The results of our preliminary study in which mo-DCs of HCs exposed to gp120 leading to the up-regulation of SOCS-1 along with reduced cytokine secretion prompted us to evaluate its expression in HIV infected cohorts at different stages of disease [39]. The assessment of SOCS-1 expression in relation to disease progression has not been contemplated so far and our findings indicate that DCs of patients in advanced stage have diminished cytokine production concurrent with elevated SOCS-1 level. Our study is in-line with another *in-vitro* study showing loss of DCs after addition of GM-CSF/IL-4 coupled with reduced capacity to secrete cytokines, lower expression of co-stimulatory molecules and an increased expression of SOCS-1/SOCS-3 [40]. Moreover we further validated our observations in a small subset of patients wherein we have shown an improvement in the functional status of DCs after siRNA mediated knocking of SOCS-1 expression, showing enhanced cytokine production and augmented allo-stimulatory capacity, which was consistent with other studies in transgenic mice and HIV seropositive patients [14]. An increased production of proinflammatory cytokines, such as IL-12 is critical for the induction of efficacious T cell-mediated immunity by significantly enhancing the potency of primary CD8+ T cell responding to HIV Gag antigens.

Further, the elevated levels of SOCS-3 and SHP-1, which are known to play an inhibitory role in cell signaling initiated either by cytokines/growth factors, was also observed coupled with lower expression of JAK2 and NF-κB1 in the patients with advanced stage of HIV-1 disease, suggested the imbalance of negative and positive regulators of cytokine signaling playing role in down-modulation of the DC functions during this stage of disease. It has been reported earlier that SOCS-1 can affect the TLR-NF-κB pathway by interacting directly with its p65 subunit, marking its ubiquitylation and degradation or suppressing NF-κB activation [41,42]. Another mechanism suggests interference of JAK-STAT signaling whereby activation of JAK2 and STAT5 by LPS involved in IL-6 induction is inhibited by SOCS-1 [43,44]. The silencing of SHP-1 has been shown to result in increased cytokine production and NF-κB activation, corresponding to increased DC survival [45]. Furthermore, in another *in-vitro* study on mo-DCs infected with adenovirus, it was shown that effective antigen presentation was dependent on NF-κB because all TLR signaling pathways culminate in its activation controlling the downstream expression of an array of cytokine genes [46].

## Conclusions

The results of our study with an extensive evaluation of phenotypic and functional aspects of DCs in human cohorts at different stages of HIV-1 disease indicate severe impairment of DC functions in the advanced stage. Based on our observations, it can be proposed that chronic TLR stimulation of DCs potently induces SOCS-1, SOCS-3 or SHP-1 which may have a profound negative effect either on the JAK-STAT or TLR-NF-κB pathways that ultimately exert inhibitory effects on cytokine production, hence down-modulating their capability to handle opportunistic infections. Our study has implications for the design of new generation preventive or therapeutic vaccines based on silencing of one or more of these negative regulators after studying the exact contribution of each of them at transcriptional level. Understanding DC dysregulation as per disease pattern will pave the way for designing of novel therapeutics through proper DC stimulation for not only blocking further dissemination of HIV but also reverting the tolerogenic environment for better handling of bacterial onslot in this stage of disease.
